# Supplementary material for: Exploring the Biological Properties of Zn(II) Bisthiosemicarbazone Helicates
Source: Int J Mol Sci. 2023 Jan 23;24(3):2246. doi: 10.3390/ijms24032246 (PMC9916454; doi:10.3390/ijms24032246)
Supplement: Supplementary file 1 [file ijms-24-02246-s001.zip › ijms-2147433-supplementary.pdf]

International Journal of *Molecular Sciences*

MDPI

*Supporting Information*

## 1. *Bis*thiosemicarbazone ligands $H_2L^{Me}$, $H_2L^{Ph}$ and $H_2L^{PhNO2}$

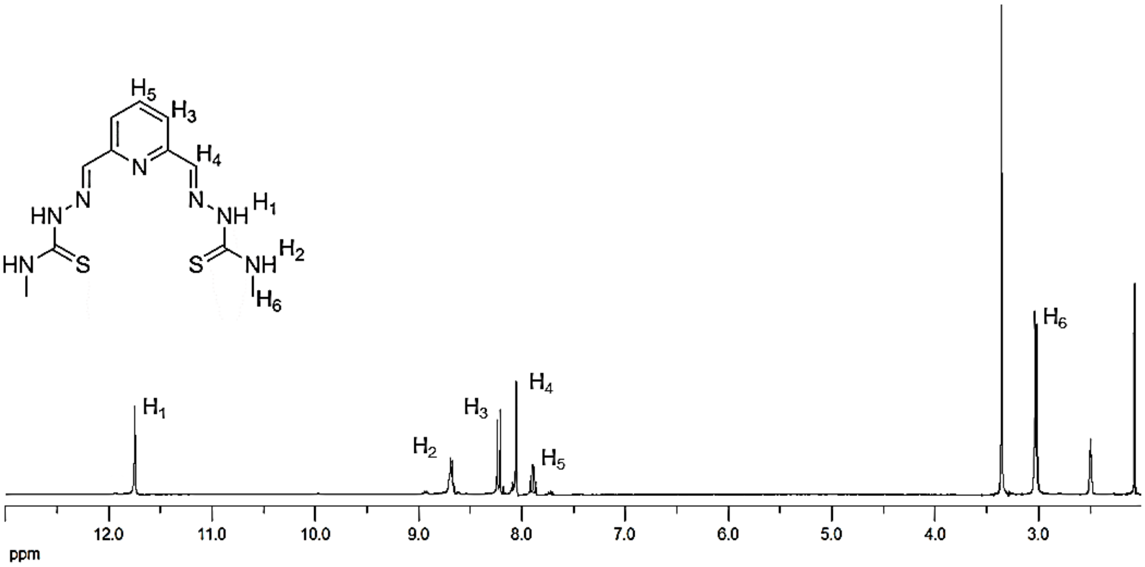


Figure S1. $^{1}$H NMR spectra of $H_2L^{Me}\cdot 2H_2O$ (DMSO-$d_6$, r.t.). **$^{1}$H NMR [DMSO-d$_6$, δ (m, nH, Hx, J)]:** 11.76 (s, 2H, $H_1$), 8.71 ($c_e$, 2H, $H_2$, J= 4.4 Hz), 8.25 (d, 2H, $H_3$, J= 7.8 Hz), 8.06 (s, 2H, $H_4$), 7.90 (t, 1H, $H_5$, J= 7.8 Hz), 3.04 (d, 6H, $H_6$, J= 4.1 Hz).

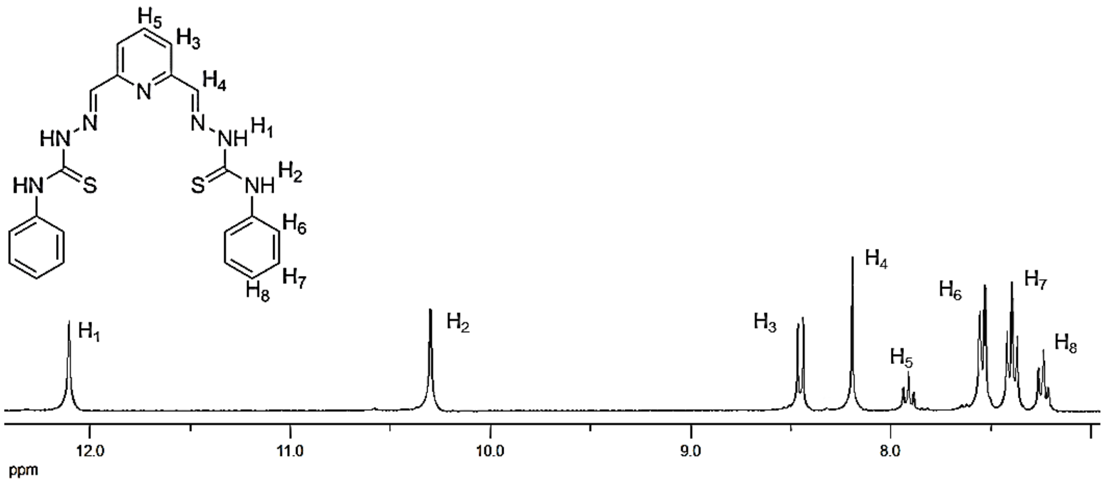


Figure S2. $^{1}$H NMR spectra of $H_2L^{Ph}\cdot 2H_2O$ (DMSO-$d_6$, r.t.). **$^{1}$H NMR [DMSO-d$_6$, δ (m, nH, Hx, J)]:** 12.11 (s, 2H, $H_1$), 10.30 (s, 2H, $H_2$), 8.46 (d, 2H, $H_3$, J= 7.8 Hz), 8.19 (s, 2H, $H_4$), 7.91 (t, 1H, $H_5$, J= 7.8 Hz), 7.56 (d, 4H, $H_6$, J= 7.6 Hz), 7.40 ($t_a$, 4H, $H_7$, $J_1$= 7.6 Hz, $J_2$= 8.0 Hz), 7.24 (t, 2H, $H_8$, $J_1$= 7.4 Hz, $J_2$= 7.3 Hz).

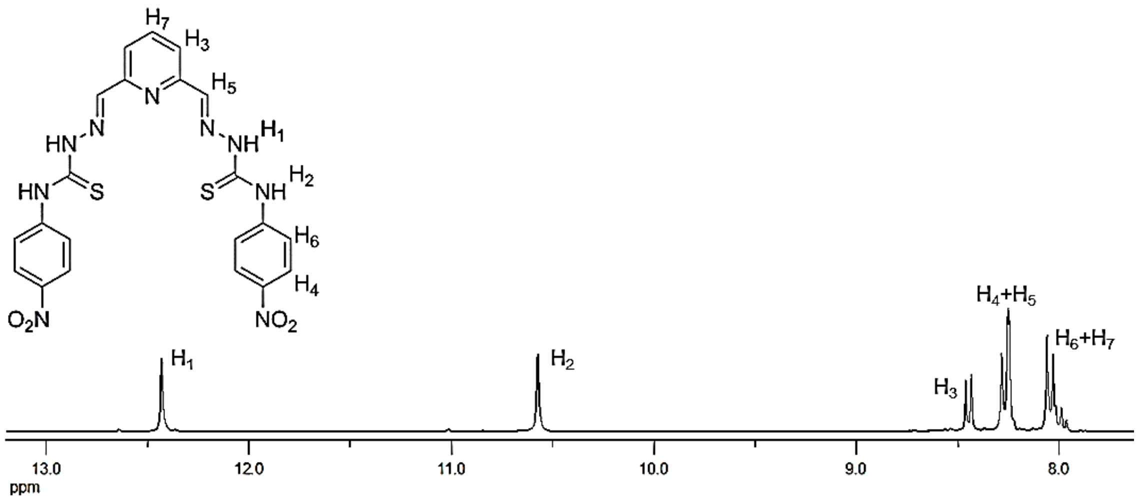


Figure S3. $^{1}$H NMR spectra of $H_2L^{PhNO2}$ (DMSO-d$_6$, r.t.). **$^{1}$H NMR [DMSO-d$_6$, δ (m, nH, Hx, J)]:** 12.43 (s, 2H, $H_1$), 10.57 (s, 2H, $H_2$), 8.46 (d, 2H, $H_3$, J= 7.8 Hz), 8.30-8.20 (m, 6H, $H_4$+$H_5$), 8.10-7.90 (m, 1H, $H_6$+$H_7$).

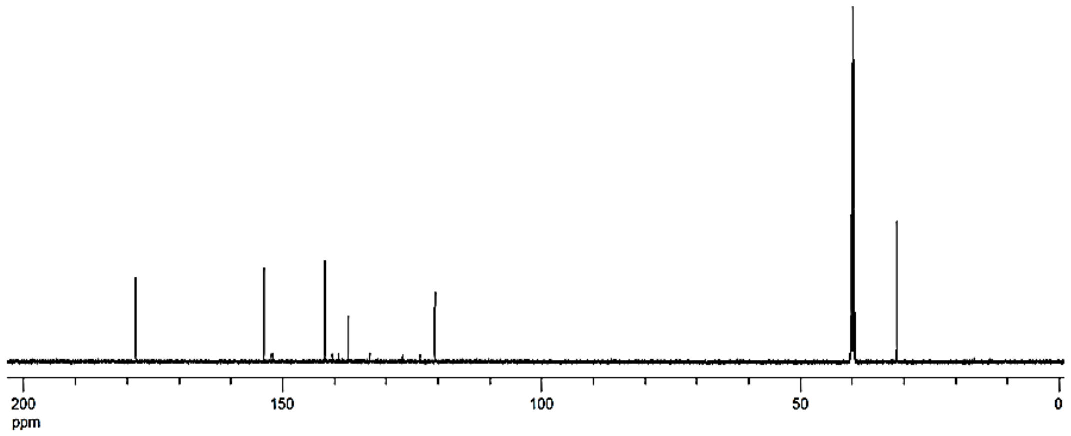


Figure S4. $^{13}$C NMR spectra of $H_2L^{Me}$·$2H_2O$ (DMSO-d$_6$, r.t.). **$^{13}$C NMR (DMSO-d$_6$, ppm):** 178.3 (C=S), 153.3 (C=N), 141.3 ($C_{ar}$), 137.5 ($CH_{ar}$), 120.6 ($CH_{ar}$), 31.26 ($CH_3$).

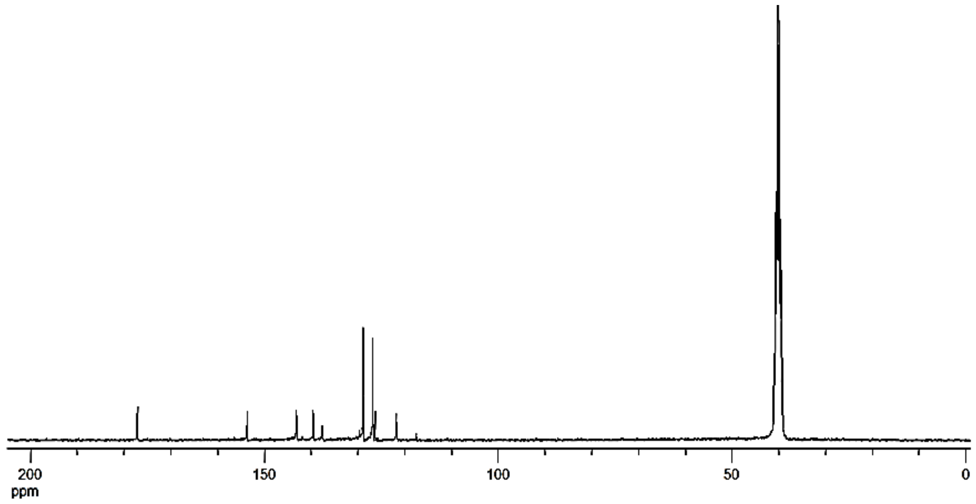


Figure S5. $^{13}$C NMR spectra of $H_2L^{Ph}$·$2H_2O$ (DMSO-d$_6$, r.t.). **$^{13}$C NMR (DMSO-d$_6$, ppm):** 177.1 (C=S), 153.7 (C=N), 143.1 ($C_{ar}$), 139.6 ($C_{ar}$), 137.7 ($CH_{ar}$), 128.8 ($CH_{ar}$), 126.8 ($CH_{ar}$), 126.3 ($CH_{ar}$), 121.8 ($CH_{ar}$).

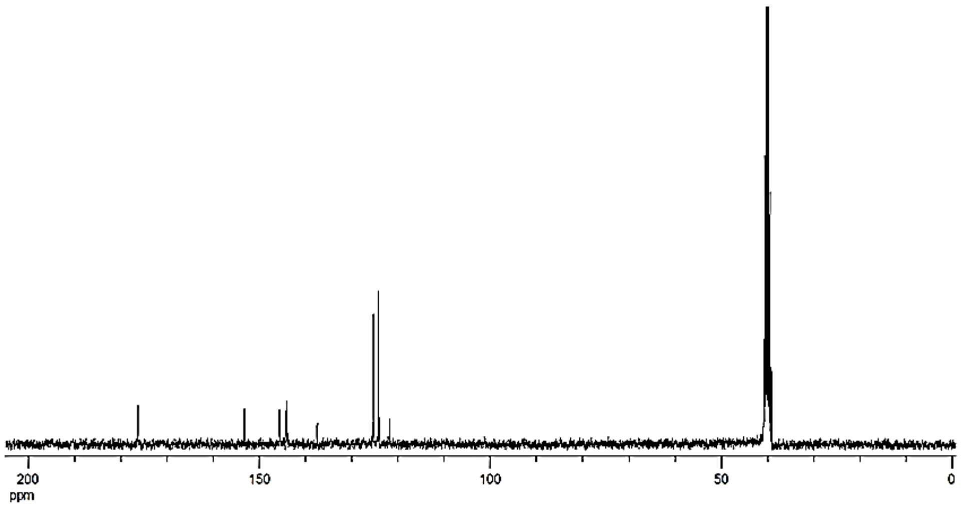


Figure S6. $^{13}C$ NMR spectra of $H_2L^{PhNO2}$ (DMSO-$d_6$, r.t.). **$^{13}C$ NMR (DMSO-$d_6$, ppm):** 176.4 (C=S), 153.3(C=N), 145.7($C_{ar}$), 144.2 ($C_{ar}$), 144.0 ($C_{ar}$), 137.5 ($CH_{ar}$), 125.3 ($CH_{ar}$), 124.2 ($CH_{ar}$), 121.8 ($CH_{ar}$).

## 2. *Bis*thiosemicarbazone derived zinc(II) dihelicates

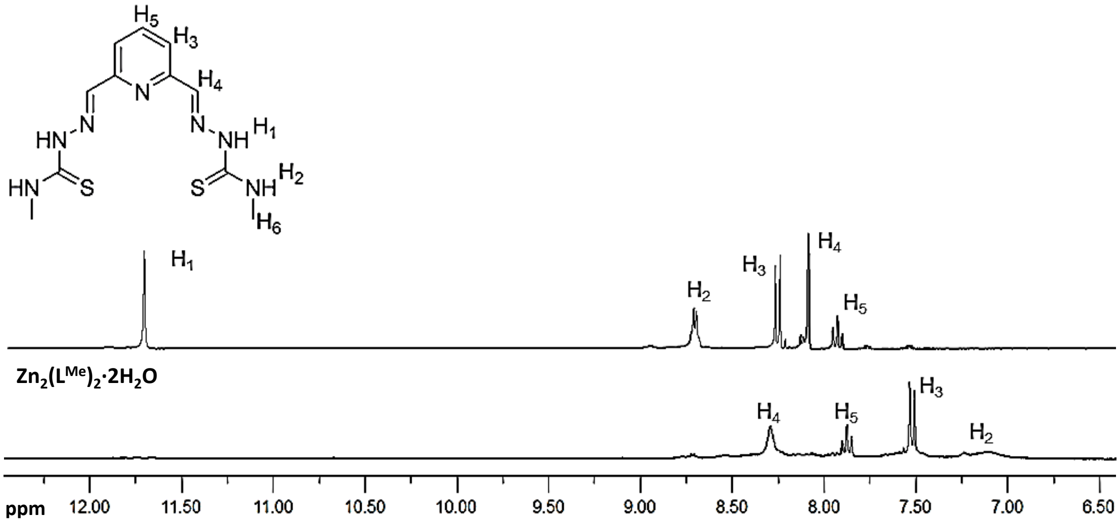


Figure S7. $^1H$ NMR spectra of $Zn_2(L^{Me})_2 \cdot 2H_2O$ (DMSO-$d_6$, r.t.). **$^1H$ NMR [DMSO-$d_6$, δ (m, nH, Hx, J)]:** 8.26 (s, 2H, $H_4$), 7.83 (t, 1H, $H_5$, J= 7.7 Hz), 7.48 (d, 2H, $H_3$, J= 7.7 Hz), 7.22 ($s_a$, 1H, $H_2$), 2.75 (d, 6H, $H_6$, J= 4.2 Hz).

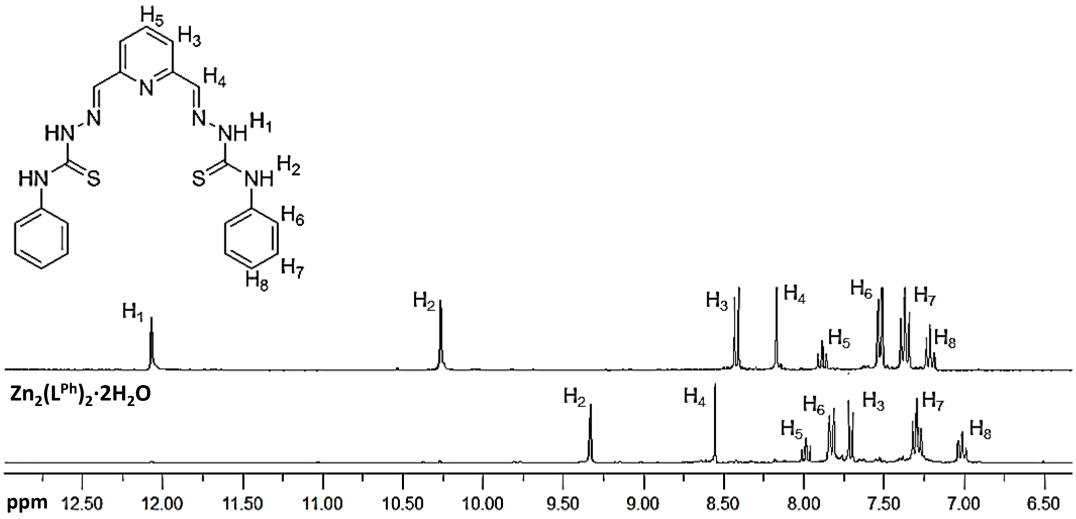

Figure S8. $^1$H NMR spectra of $Zn_2(L^{Ph})_2{\cdot}2H_2O$ (DMSO-$d_6$, r.t.). **$^1$H NMR [DMSO-d$_6$, δ (m, nH, Hx, J)]:** 9.34 (s, 2H, $H_2$), 8.56 (s, 2H, $H_4$), 7.99 (d, 1H, $H_5$, J= 7.8 Hz), 7.84 (d, 4H, $H_6$, J= 7.8 Hz), 7.70 (d, 2H, $H_3$, J= 7.8 Hz), 7.30 ($t_a$, 4H, $H_7$, $J_1$= 7.8 Hz, $J_2$= 8.0 Hz), 7.01 ($t_a$, 2H, $H_8$, $J_1$= 7.4 Hz, $J_2$= 6.9 Hz).

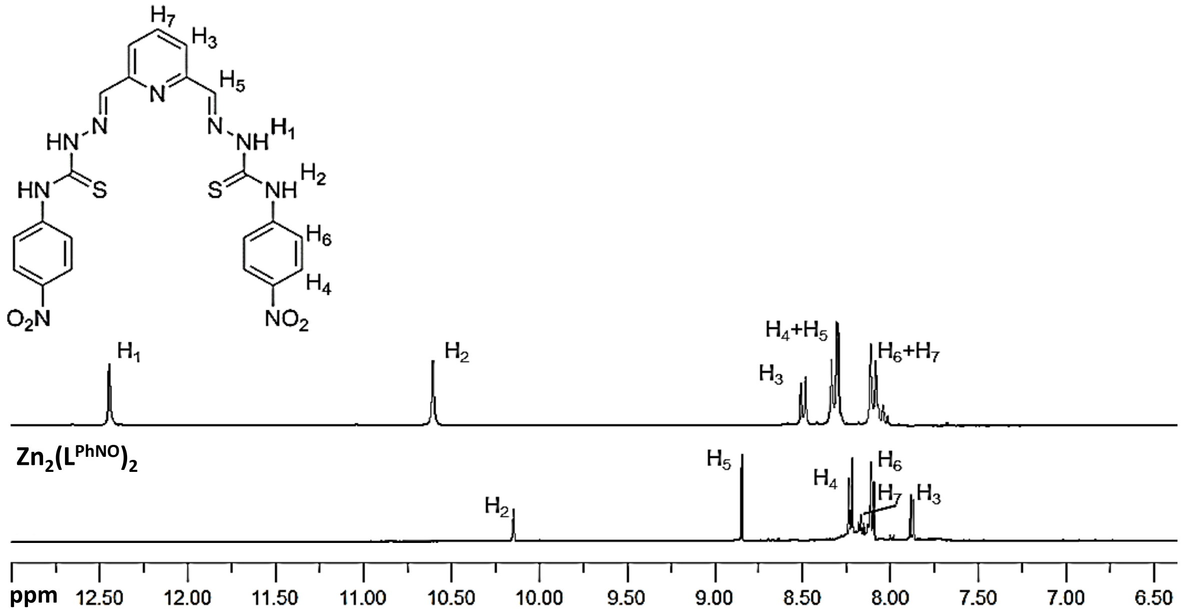


Figure S9. $^1$H NMR spectra of $Zn_2(L^{PhNO2})_2$ (DMSO-$d_6$, r.t.). **$^1$H NMR [DMSO-d$_6$, δ (m, nH, Hx, J)]:** 10.11 (s, 2H, $H_2$), 8.80 (s, 2H, $H_5$), 8.17 (d, 1H, $H_4$, J= 7.7 Hz), 8.12 (t, 1H, $H_7$, J= 7.7 Hz), 8.05 (d, 4H, $H_6$, J= 9.3 Hz), 7.82 (d, 2H, $H_3$, J= 7.7 Hz).

Table S1. Main crystallographic data for helicates $[Zn_2(L^{Me})_2]\cdot 3H_2O$ **1***, $[Zn_2(L^{Ph})_2]\cdot 4CH_3CN$ **2*** and $[Zn_2(L^{PhNO2})_2]$ **3***.

| | **$[Zn_2(L^3\text{-Me})_2]\cdot 3H_2O$** | **$[Zn_2(L^3\text{-Ph})_2]\cdot 4CH_3CN$** | **$[Zn_2(L^3\text{-PhNO}_2)_2]$** |
|---|---|---|---|
| **Molecular formula** | $Zn_2C_{22}H_{32}N_{14}S_4$ | $Zn_2C_{50}H_{46}N_{18}S_4$ | $Zn_2C_{42}H_{30}N_{18}O_8S_4$ |
| **Molecular weight** | 799.68 | 1158.03 | 1173.82 |
| **Spatial group** | *I2/a* | P -1 | *C2/c* |
| **Crystalline system** | Monoclinic | Triclinic | Monoclinic |
| **Crystal size/mm** | 0.30 × 0.07 × 0.05 | 0.18 × 0.14 × 0.04 | 0.8 × 0.2 × 0.09 |
| **a/Å** | 14.424(4) | 10.6496(5) | 21.5284(13) |
| **b/Å** | 12.7407(17) | 15.3841(9) | 14.0991(9) |
| **c/Å** | 16.748(2) | 18.3830(10) | 15.6869(8) |
| **α/º** | 90 | 97.235(2) | 90 |
| **β/º** | 99.930(7) | 105.836(2) | 94.982(4) |
| **γ/º** | 90 | 108.760(3) | 90 |
| **Temperature/K** | 100 | 373(2) | 100 |
| **Volume/Å$^3$** | 3031.6(10) | 2667.0(2) | 4743.5(5) |
| **Z** | 4 | 2 | 4 |
| **Density/g cm$^{-3}$** | 1.752 | 1.442 | 1.644 |
| **Intervale θ/º** | 2.5 - 19.1 | 2.4 - 21.6 | 2.9 - 26.2 |
| **Measured reflexions** | 23453 | 40098 | 24181 |
| **Unique reflexions [$R_{int}$]** | 2896 [0.150] | 10094 [0.086] | 3478 [0.054] |
| **μ/mm$^{-1}$** | 1.91 | 1.110 | 1.262 |
| **F(000)** | 1640 | 1192 | 2384 |
| **Residues/e Å$^{-3}$** | 0.56 and -0.94 | 0.794 and -0.537 | 1.18 and -0.63 |
| **R Final Index [I>2sigma(I)]** | 0.0612 [0.1383] | 0.0545 [0.1138] | 0.0342 [0.0779] |
| **R Index [all data]** | 0.1108 [0.1682] | 0.1038 [0.1298] | 0.0522 [0.0845] |

Table S2. Main bond distances and angles for $[Zn_2(L^{Me})_2]\cdot 3H_2O$ **1*** zinc helicate.

| **Bond distances (Å)** | | | | | |
|---|---|---|---|---|---|
| N(3)-Zn(1) | 2.114(5) | S(3)-Zn(1) | 2.3865(19) | C(9)-N(5) | 1.263(8) |
| N(5)-Zn(1) | 2.124(5) | C(3)-N(3) | 1.290(8) | C(10)-S(2) | 1.747(7) |
| N(4)-Zn(1) | 2.485(5) | C(2)-S(3) | 1.739(6) | S(2)-Zn(1) | 2.3423(19 |
| **Bond angles (º)** | | | | | |
| N(3)-Zn(1)-S(3) | 80.53(15) | N(3)-Zn(1)-N(4) | 70.94(18) | C(9)-N(5)-N(6) | 116.0(5) |
| N(5)-Zn(1)-S(2) | 82.37(15) | N(5)-Zn(1)-N(4) | 102.64(18) | C(10)-N(5)-N(6) | 112.8(5) |
| S(3)-Zn(1)-S(2) | 112.48(7) | C(3)-N(3)-N(2) | 116.3(5) | N(1)-C(2)-S(3) | 115.7(5) |
| C(2)-N(2)-N(3) | 113.2(5) | | | | |

Table S3. Main bond distances and angles for $[Zn_2(L^{Ph})_2]\cdot 4CH_3CN$ **2*** zinc helicate.

| **Bond distances (Å)** | | | | | |
|---|---|---|---|---|---|
| N(3)-Zn(1) | 2.081(4) | S(1)-Zn(1) | 2.3593(12) | C(14)-N(5) | 1.280(6) |
| N(4)-Zn(1) | 2.463(3) | S(2)-Zn(2) | 2.3381(13) | C(15)-S(2) | 1.741(5) |
| N(5)-Zn(2) | 2.081(4) | S(3)-Zn(1) | 2.3384(13) | C(28)-S(3) | 1.741(5) |
| N(10)-Zn(1) | 2.084(4) | S(4)-Zn(2) | 2.3777(12) | C(29)-N(10) | 1.284(5) |
| N(11)-Zn(2) | 2.446(3) | C(7)-S(1) | 1.738(5) | C(35)-N(12) | 1.287(5) |
| N(12)-Zn(2) | 2.080(4) | C(8)-N(3) | 1.278(5) | | |
| **Bond angles (⁰)** | | | | | |
| N(10)-Zn(1)-S(3) | 83.30(11) | N(5)-Zn(2)-S(2) | 83.58(11) | N(13)-N(12)-Zn(2) | 123.2(3) |
| N(3)-Zn(1)-S(1) | 81.96(10) | N(12)-Zn(2)-S(4) | 81.23(10) | C(36)-N(13)-N(12) | 112.7(4) |
| S(3)-Zn(1)-S(1) | 111.71(5) | S(2)-Zn(2)-S(4) | 110.53(5) | C(35)-N(12)-N(13) | 116.1(4) |
| N(3)-Zn(1)-N(4) | 72.07(13) | N(12)-Zn(2)-N(11) | 72.20(13) | N(14)-C(36)-S(4) | 114.4(3) |
| N(10)-Zn(1)-N(4) | 94.92(13) | N(5)-Zn(2)-N(11) | 97.47(13) | C(14)-N(5)-N(6) | 115.4(4) |
| C(8)-N(3)-N(2) | 116.7(4) | C(29)-N(10)-N(9) | 116.0(4) | C(15)-N(6)-N(7) | 117.3(4) |
| C(7)-N(2)-N(3) | 112.7(4) | C(28)-N(9)-N(10) | 113.3(4) | N(7)-C(15)-S(2) | 114.3(4) |
| N(1)-C(7)-S(1) | 94.97(16) | N(8)-C(28)-S(3) | 113.6(3) | | |

Table S4. Main bond distances and angles for $[Zn_2(L^{PhNO2})_2]$ **3*** zinc helicate.

| **Bond distances (Å)** | | | | | |
|---|---|---|---|---|---|
| N(4)-Zn(1) | 2.062(2) | S(3)-Zn(2) | 2.3857(7) | C(14)-N(6) | 1.287(3) |
| S(4)-Zn(1) | 2.3203(7) | C(7)-S(4) | 1.737(3) | C(15)-S(3) | 1.737(3) |
| N(6)-Zn(2) | 2.116(2) | C(8)-N(4) | 1.279(3) | | |
| **Bond angles (⁰)** | | | | | |
| N(4)-Zn(1)-S(4) | 84.14(6) | N(6)-Zn(2)-S(3) | 110.45(6) | C(8)-N(4)-N(3) | 115.1(2) |
| S(4)-Zn(1)-S(4) | 122.23(4) | S(3)-Zn(2)-S(3) | 118.20(4) | C(14)-N(6)-N(7) | 114.5(2) |
| N(4)-Zn(1)-N(4) | 145.32(12) | N(6)-Zn(2)-N(6) | 158.14(12) | N(3)-C(7)-S(4) | 127.9(2) |
| N(7)-C(15)-S(3) | 127.7(2) | N(2)-C(7)-N(3) | 117.3(2) | N(7)-C(15)-N(8) | 116.6(2) |

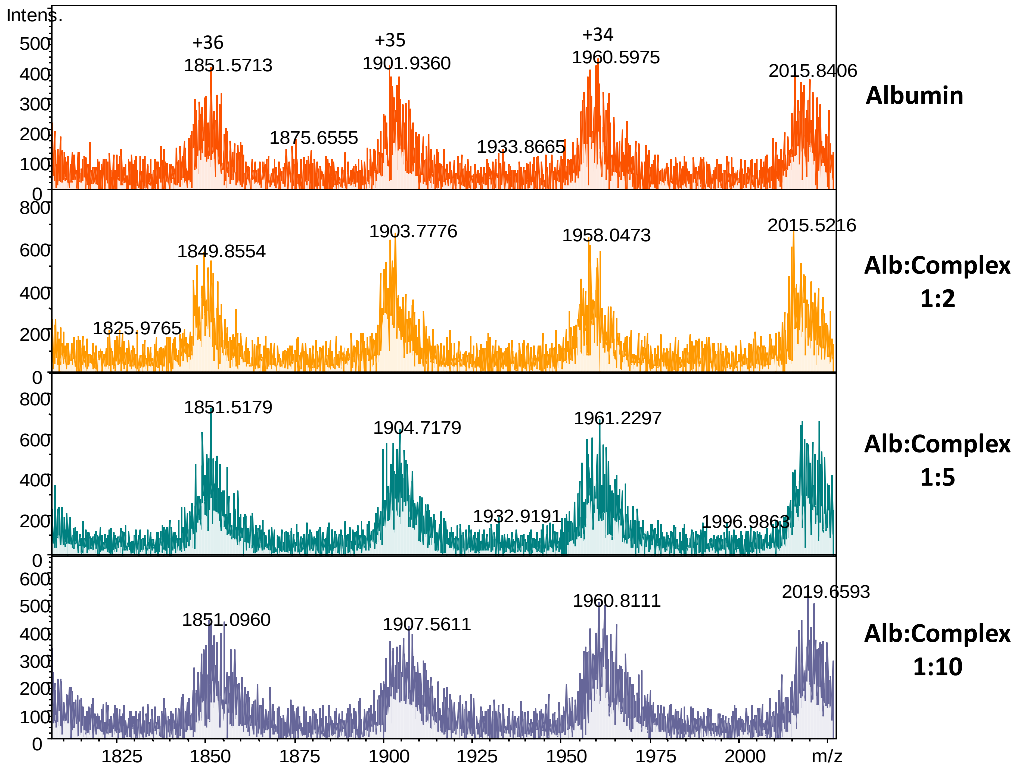


Figure S10. ESI MS-TOF titration of human serum albumin (MW= 66550 Da) with $Zn_2(L^{Ph})_2 \cdot 2H_2O$ **2** helicate.

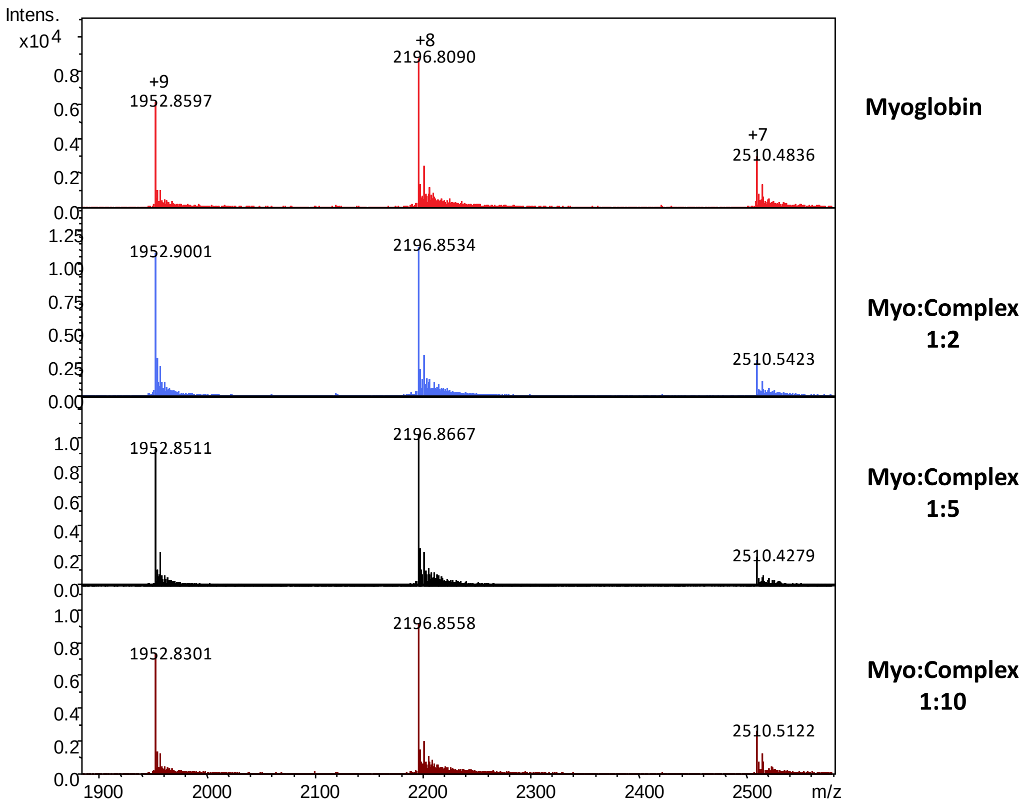


Figure S11. ESI MS-TOF titration of myoglobin (MW= 17567 Da) with $Zn_2(L^{Ph})_2 \cdot 2H_2O$ **2** helicate.

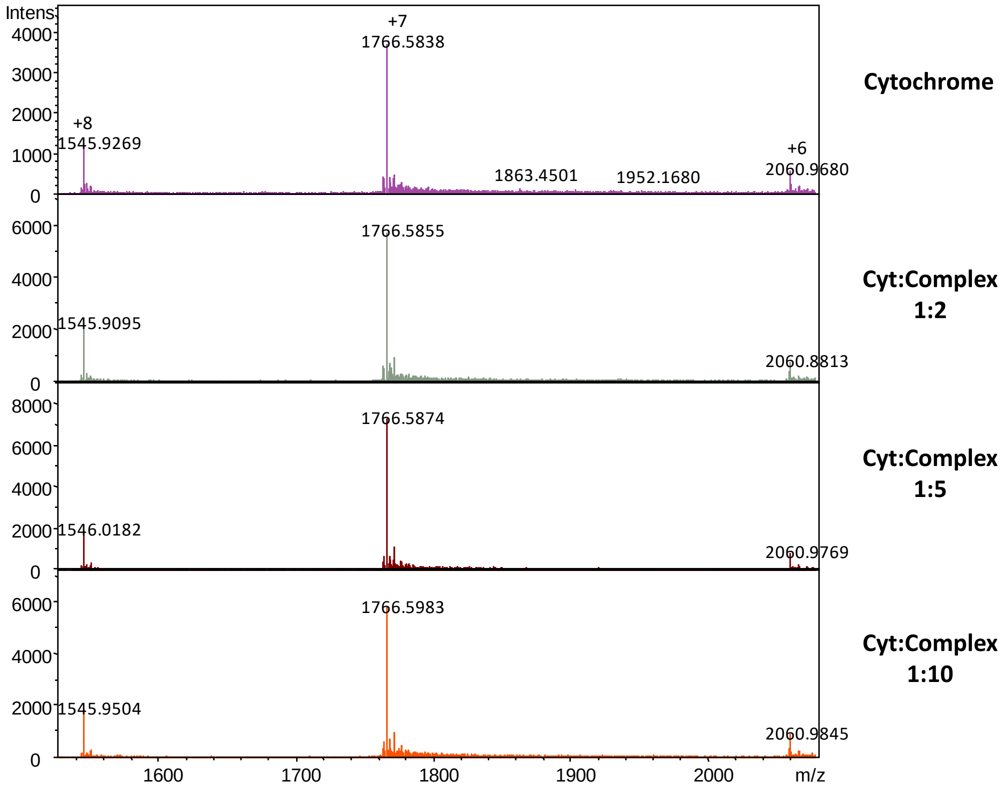


Figure S12. ESI MS-TOF titration of cytochrome C (MW= 12359 Da) with $Zn_2(L^{Ph})_2{\cdot}2H_2O$ **2** helicate.

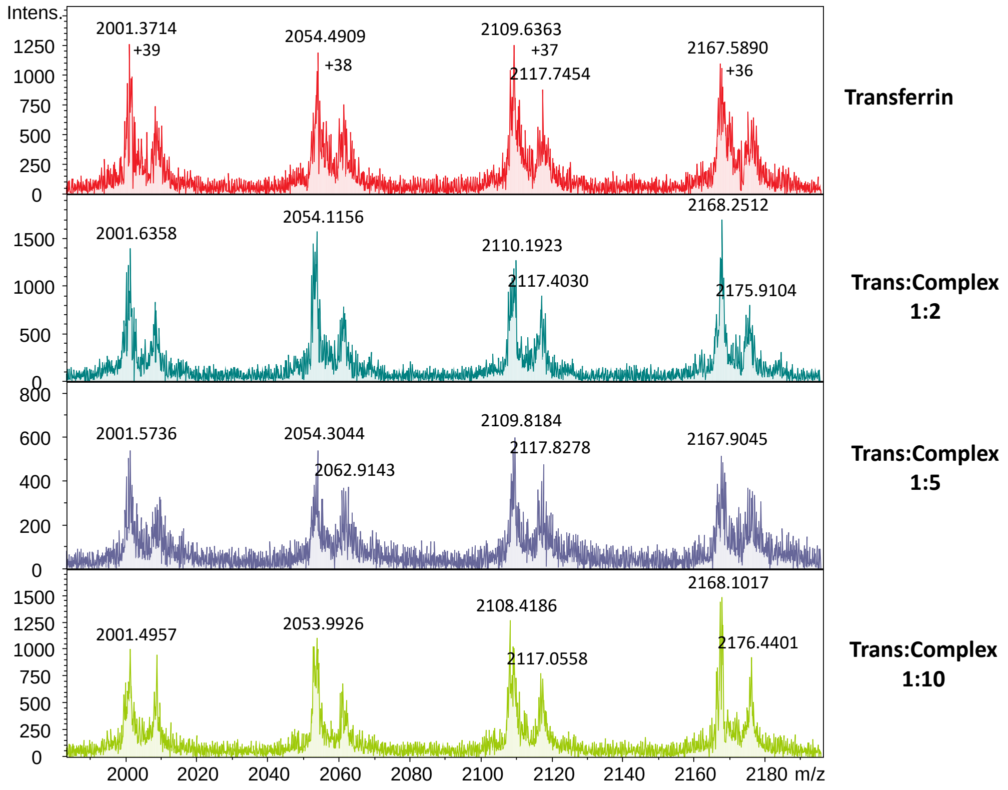


Figure S13. ESI MS-TOF titration of transferrin (MW= 78019 Da) with $Zn_2(L^{Ph})_2{\cdot}2H_2O$ **2** helicate.

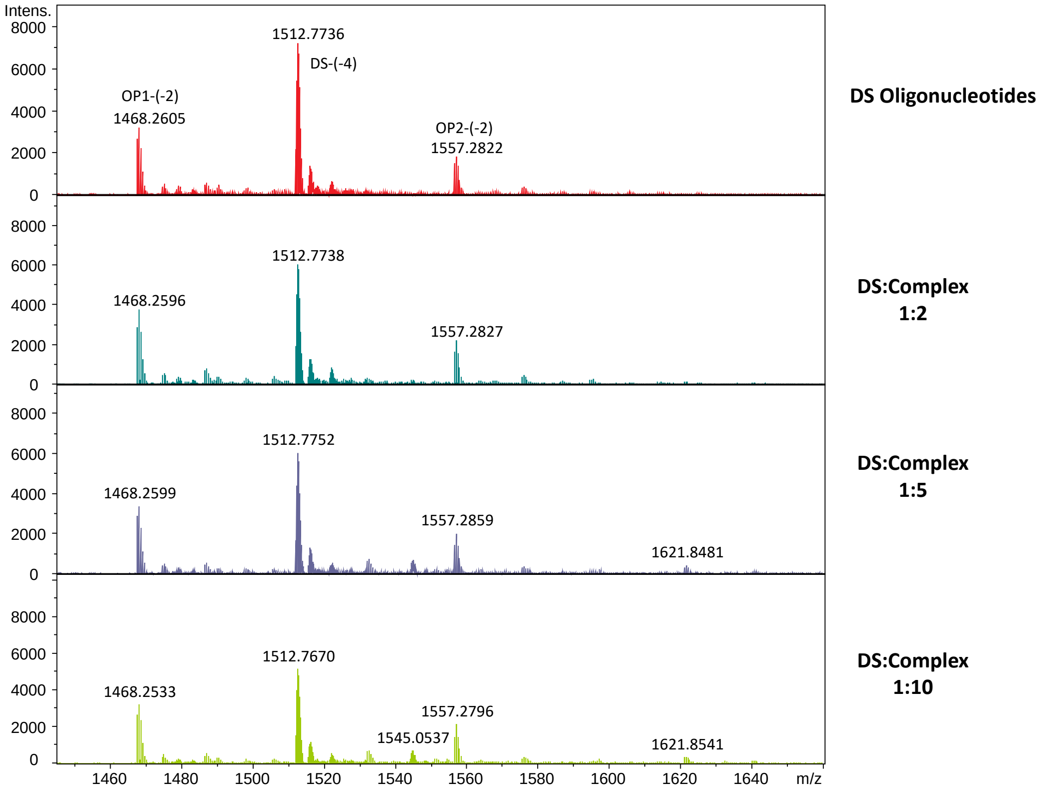


Figure S14. ESI MS-TOF titration of double-stranded oligonucleotides (DS) with $Zn_2(L^{Ph})_2{\cdot}2H_2O$ **2** helicate.

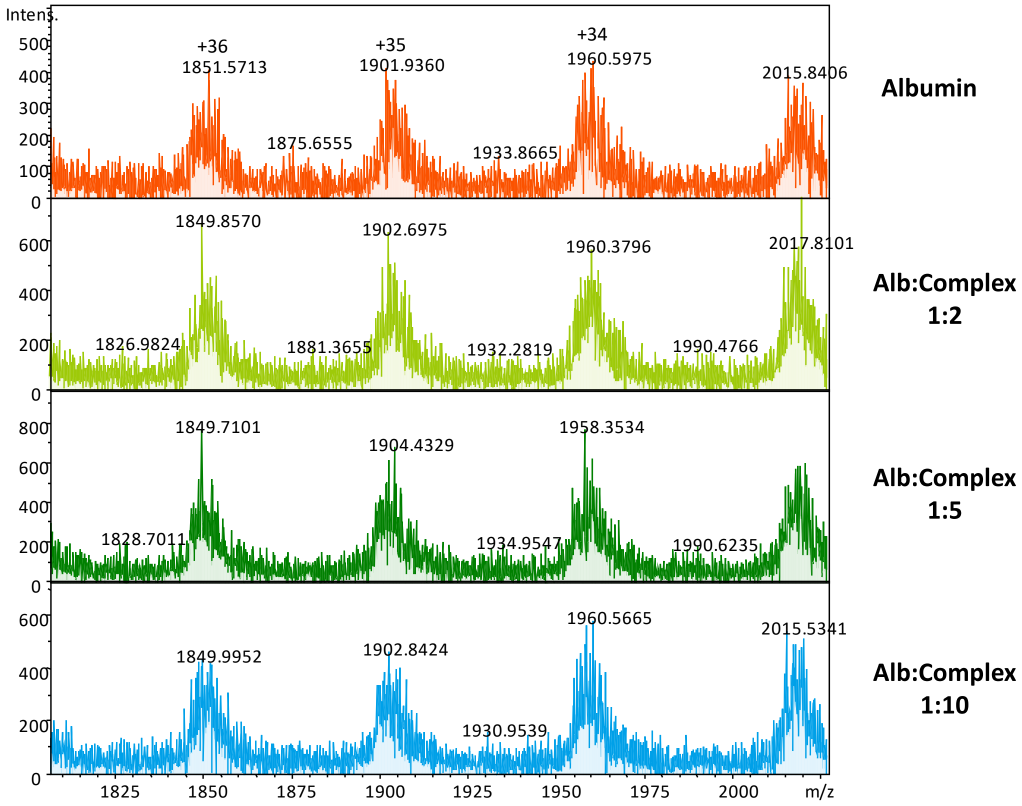


Figure S15. ESI MS-TOF titration of human serum albumin (MW= 66550 Da) with $Zn_2(L^{PhNO2})_2$ **3** helicate.

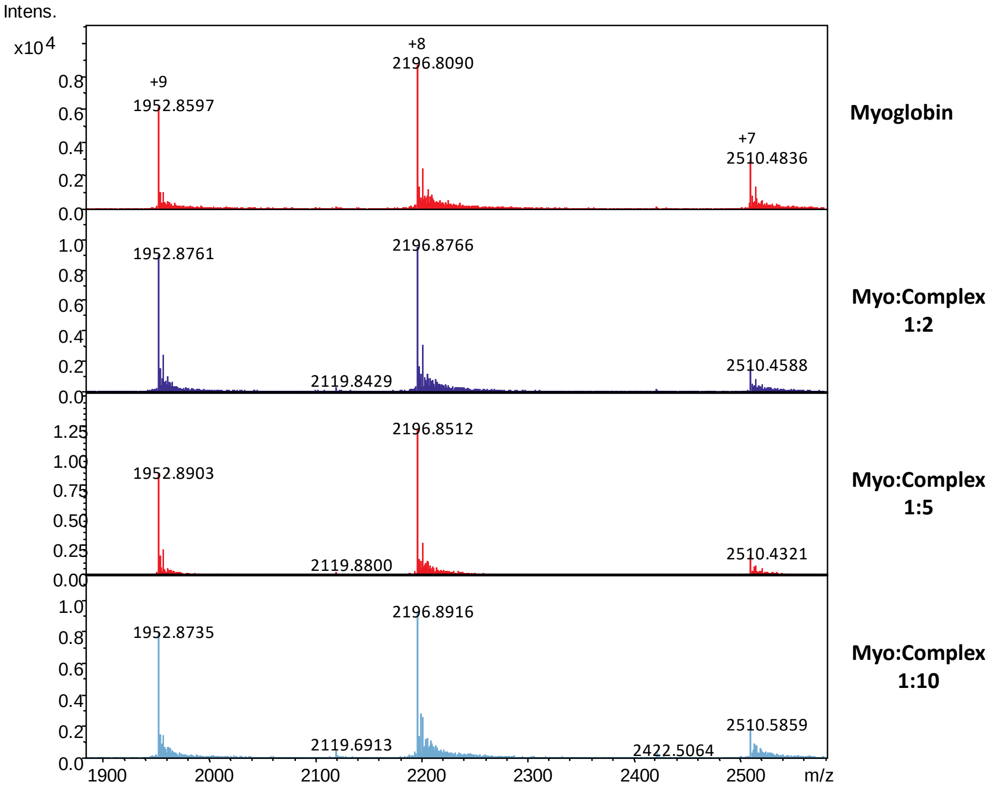


Figure S16. ESI MS-TOF titration of myoglobin (MW= 17567 Da) with $Zn_2(L^{PhNO2})_2$ **3** helicate.

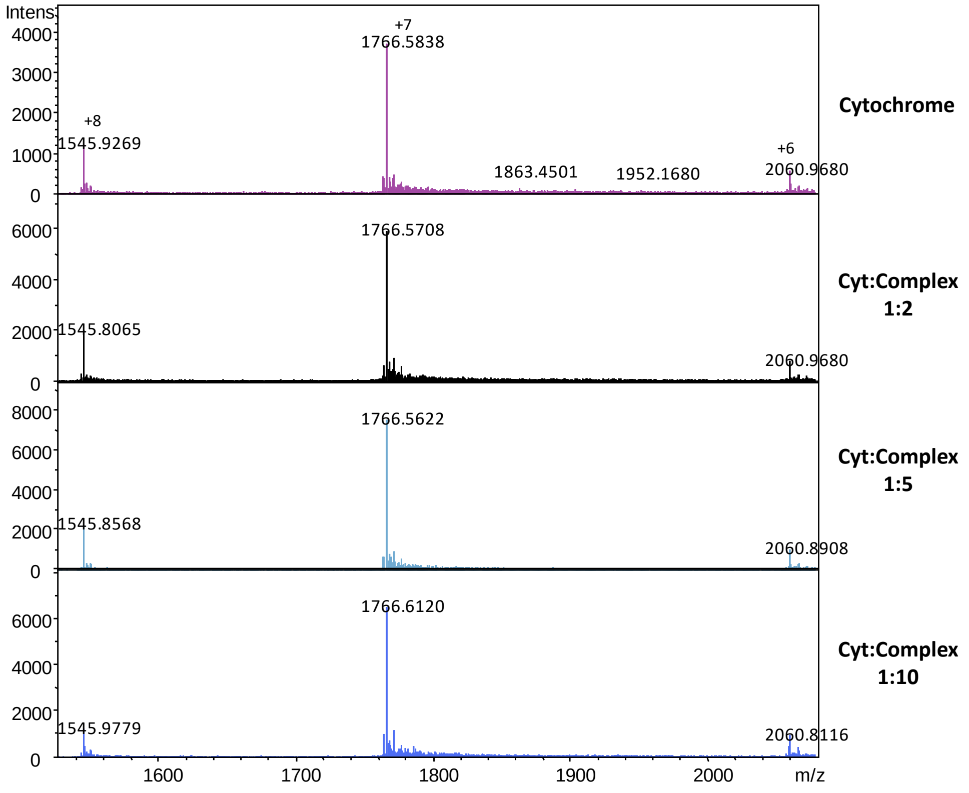


Figure S17. ESI MS-TOF titration of cytochrome C (MW= 12359 Da) with $Zn_2(L^{PhNO2})_2$ **3** helicate.

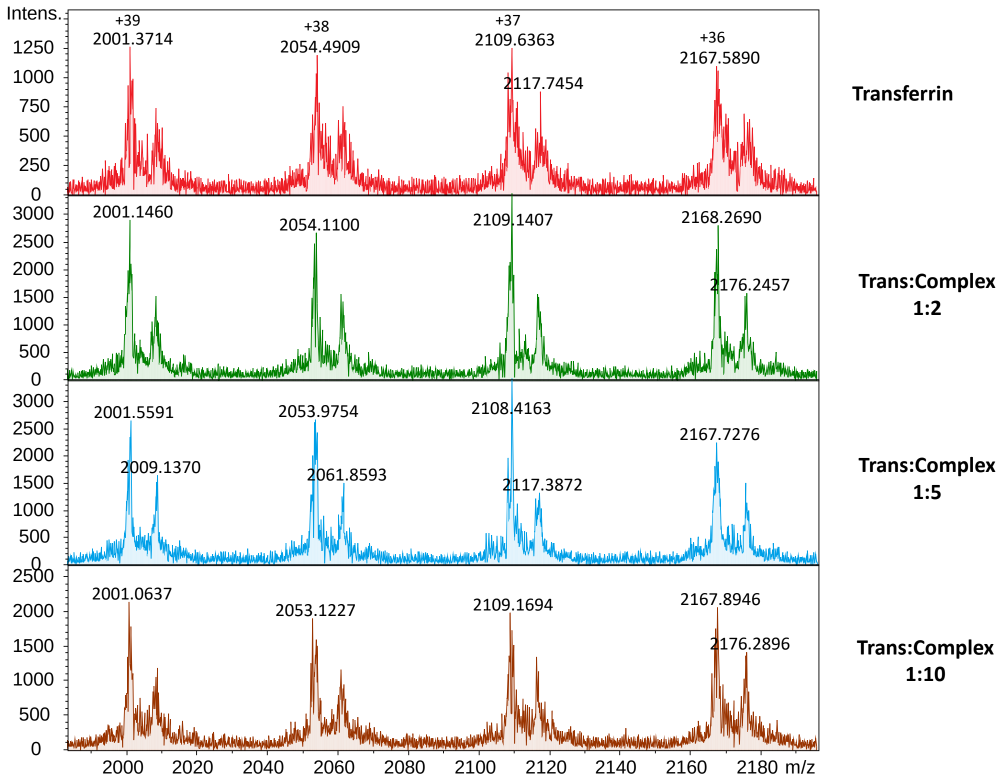


Figure S18. ESI MS-TOF titration of transferrin (MW= 78019 Da) with $Zn_2(L^{PhNO2})_2$ **3** helicate.

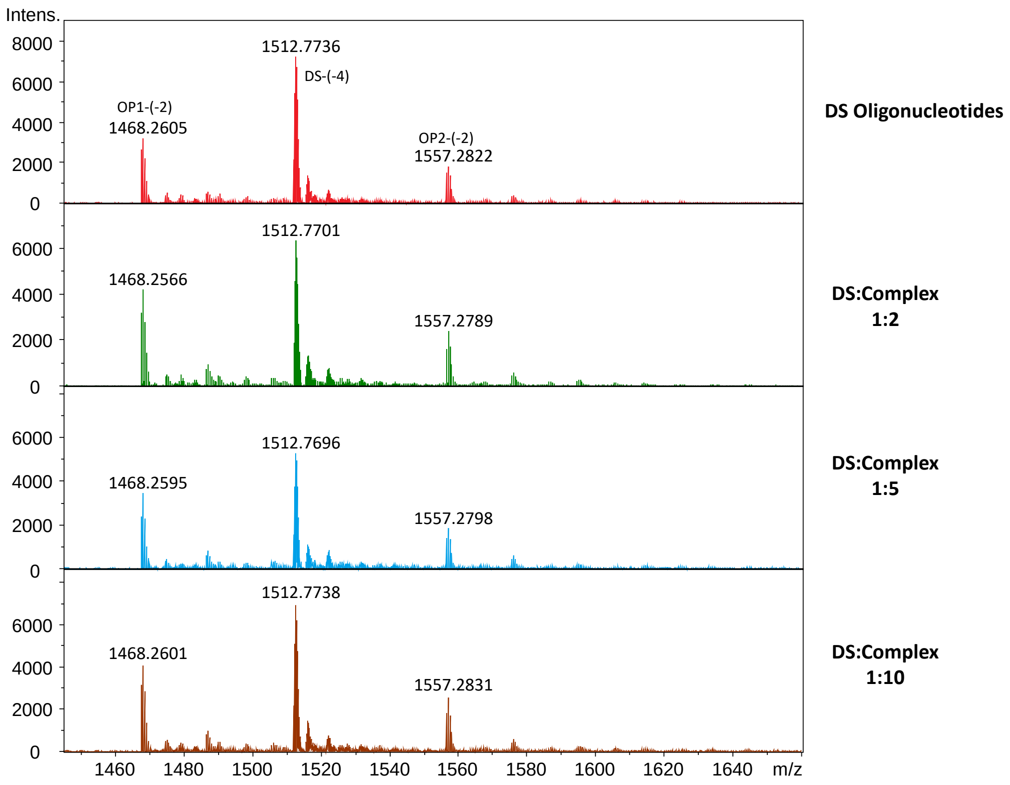


Figure S19. ESI MS-TOF titration of double-stranded oligonucleotides (DS) with $Zn_2(L^{PhNO2})_2$ **3** helicate.
